# Validation of clinical risk tools for recurrent *Clostridioides difficile* infection

**DOI:** 10.1017/ice.2024.75

**Published:** 2024-09

**Authors:** Rachel H. Boone, Emmanuel Lee, William A. Petri, Gregory R. Madden

**Affiliations:** 1 Department of Microbiology, Immunology, and Cancer Biology, University of Virginia, Charlottesville, VA, USA; 2 University of Virginia School of Medicine, Charlottesville, VA, USA; 3 Division of Infectious Diseases & International Health, Department of Medicine, University of Virginia School of Medicine, Charlottesville, VA, USA

## Abstract

**Objective:**

We sought to validate available tools for predicting recurrent *C. difficile* infection (CDI) including recurrence risk scores (by Larrainzar-Coghen, Reveles, D’Agostino, Cobo, and Eyre *et al*) alongside consensus guidelines risk criteria, the leading severity score (ATLAS), and PCR cycle threshold (as marker of fecal organism burden) using electronic medical records.

**Design:**

Retrospective cohort study validating previously described tools.

**Setting:**

Tertiary care academic hospital.

**Patients:**

Hospitalized adult patients with CDI at University of Virginia Medical Center.

**Methods:**

Risk scores were calculated within ±48 hours of index CDI diagnosis using a large retrospective cohort of 1,519 inpatient infections spanning 7 years and compared using area under the receiver operating characteristic curve (AUROC) and the DeLong test. Recurrent CDI events (defined as a repeat positive test or symptom relapse within 60 days requiring retreatment) were confirmed by clinician chart review.

**Results:**

Reveles *et al* tool achieved the highest AUROC of 0.523 (and 0.537 among a subcohort of 1,230 patients with their first occurrence of CDI), which was not substantially better than other tools including the current IDSA/SHEA C. difficile guidelines or PCR cycle threshold (AUROC: 0.564), regardless of prior infection history.

**Conclusions:**

All tools performed poorly for predicting recurrent *C. difficile* infection (AUROC range: 0.488–0.564), especially among patients with a prior history of infection (AUROC range: 0.436–0.591). Future studies may benefit from considering novel biomarkers and/or higher-dimensional models that could augment or replace existing tools that underperform.

## Introduction

*Clostridioides difficile* is a Gram positive, spore forming, toxin producing bacterium which can colonize and infect the human intestinal track causing a robust immune response.^1^ Unlike most other infectious diseases, *C. difficile* infection (CDI) tends to relapse and re-infect despite antibiotic treatment and remission of symptoms. *C. difficile* spores are capable of surviving treatment, resulting in persistent carriage, serving as a nidus for recurrent infection. Most recurrent CDI episodes occur within 2–8 weeks of the initial infection and are due to the previous strain.^2^ Newer anti-CDI therapies (ie, fidaxomicin,^3^ bezlotoxumab,^4^ and fecal microbiota transplant^5^) effectively prevent recurrent CDI and as of April 2023,^6^ the first orally administered fecal microbiota product was approved for the prevention of recurrent CDI by the US Food and Drug Administration. However, newer therapies to prevent recurrent CDI have historically been underutilized,^7^ despite adoption by recent CDI consensus guidelines.^8^ This is likely due to two major issues with CDI treatments: first, these treatments have a high cost and logistical challenges, limiting their use to patients believed to be most at risk of recurrence,^9,10^; second, determining which patients are likely to recur is difficult without a widely accepted risk prediction model for recurrent *C. difficile* infection.

Several published outcome models (Table 1) for risk stratifying patients with CDI for developing future recurrent infection have been developed retrospectively or by using previous clinical trial data. Few recurrent CDI models have been externally validated and limited evidence suggest that they generalize poorly between centers. In addition, recently updated clinical management guidelines for *C. difficile* infection mention specific risk factors for recurrence, however, the performance of these features together as a risk stratification tool has not been externally evaluated.^8^ We were also interested in comparing *C. difficile* PCR cycle threshold (as an inverse measure of fecal organism burden), which we recently showed may be a potentially useful biomarker for recurrent infection^21^ alongside existing clinical models. Here, we validate and compare the relative performance of the leading recurrence risk models available for hospitalized cases of *C. difficile* infection.

**Table 1.** Clinical risk models for predicting recurrent *C. difficile* infection

Model name	Study context	Derivation cohort size (% with recurrence)	Definition of recurrence: time after completion of CDI treatment	Features included	AUROC: derivation cohort (validation studies)	Current study		
						AUROC: No previous CDI	AUROC: previous CDI	AUROC: all [95% CI]
Cobo^11^	Prospective: Multisite	274 (26)	2 months	Age, CDI within 1 year prior, Persistent diarrhea after 5 days, Toxin in stool	0.72 (0.43^12^)	0.502	0.496	0.522 [0.501, 0.542]
Eyre^13^	Retrospective: Multisite	1678 (20)	4 months	Age, Stool frequency ≥ 3/day, Hospital stay 4-12 weeks prior, Duration of hospital visit, Pas gastroenterology admission, Emergency admission, Emergency admission and MRSA, Emergency admission and/or dialysis/chemotherapy, Community onset, Concentration of c-reactive protein, Community onset and previous MRSA		0.521	0.466	0.515 [0.483. 0.548]
D’Agostino^14^	Clinical Trial Adapted: Antibiotic Use	1105 (18.6)	28 days	Age, Creatinine concentration, Unformed bowel movements, Prior CDI	0.64	0.482	0.458	0.488 [0.458 0.519]
Reveles^15^	Retrospective: Multisite	7538 (16)	60 days	Prior antibiotic use, PPI use, Community onset, Antidiarrheals, CDI severity		0.537	0.465	0.523 [0.490, 0.555]
Larrainzar-Coghen^16^	Retrospective: Tertiary Care Center	501 (12)	8 weeks	Age, WBC, PPI use, Enteral nutrition	0.66 (0.42^12^)	0.506	0.460	0.496 [0.464, 0.528]
Hu^17^	Retrospective: Tertiary Care Center	44 (50)	60 days	Age, Horn index, Antibiotic use after end of CDI treatment	0.80			
Zilberberg^18^	Retrospective: Tertiary Care Center	4196 (10.1)	42 days	Age, Community onset of health-care facility associated, Hospital stay within prior 60 days, Gastric acid suppression at onset, Antibiotic use at onset, Fluoroquinolone use at onset, ICU at onset	(0.591)^19^			
ATLAS^20^	Clinical Trial Adapted: Antibiotic Use	967		Age, Systemic antibiotics during CDI, WBC, Creatinine concentration, Serum albumin concentration		0.509	0.436	0.494 [0.460, 0.528]
IDSA^7^				Age, Previous CDI within 6 months, Immunocompromised host, Severe CDI		0.522	0.472	0.520 [0.487, 0.553]
PCR Ct				PCR Ct		0.578	0.591	0.564 [0.530, 0.598]

C. difficile infection (CDI), methicillin-resistant S. aureus (MRSA), proton pump inhibitor (PPI), white blood cell (WBC)

## Methods

### Study population

A previously described^22,23^ retrospective cohort of hospitalized adult patients with *C. difficile* infection at University of Virginia Medical Center (a 645-bed, tertiary care academic hospital) was used for this analysis. Hospitalized CDI cases were enrolled with a positive *C. difficile* polymerase chain reaction (PCR; GeneXpert®; Cepheid, Sunnyvale, CA) result and anti-*C. difficile* treatment. Children <18 years and episodes that were not treated with anti-*C. difficile* therapy (oral vancomycin, fidaxomicin, and/or IV/oral metronidazole) were excluded. Recurrent *C. difficile* infection was defined as the presence of a repeat positive PCR test available within 11–60 days post-infection or symptom relapse requiring retreatment/initial treatment extension for CDI within 60 days of index diagnosis. Patients without electronically available follow-up positive testing underwent additional clinician chart review (E.L., including clinician notes) to determine reports of positive *C. difficile* testing from outside laboratories and/or CDI symptom recurrence with retreatment/initial treatment extension. This study received approval from the University of Virginia Institutional Review Board (#20082).

### Data collection/risk score calculation

To identify risk models for recurrent CDI, a literature search using medical literature databases (ie, PubMed, Google Scholar) and the bibliographies of relevant manuscripts was conducted. Manuscripts were evaluated for relevance; specifically, studies which generated clinically relevant tools for predicting recurrent CDI were of interest. So that each model could be compared in later analyses, the models had to include parameters that could be reliably gathered or imputed from the electronic medical record, and >3 ordinal scores to fit to an ROC curve. Criteria used to exclude studies from further analysis included highly specific study populations (ie, children, transplant patients), single binary predictors of recurrence, multivariable logistic regression models without a clinically applicable prediction tool (ie, integer-based scoring or similar systems by which a clinician could easily estimate relative risk), use of non-clinically accessible features, and meta-analysis and reviews. 7 models were identified which fulfilled the criteria.^11,13–18^ Of these, 2 models were excluded due to inability to reproduce with our cohort (ie, Hu *et al* model included Horn Index, a subjective measure which was not among the cohort’s available features)^17^ or a lack of sufficient details for a scoring system that could be easily imputed from the electronic medical record (ie, Zilberberg *et al*).^18^ Two models, Cobo and D’Agostino, utilized definitions of recurrence (4 months and 28 days, respectively) than other models. In this study, 60 days was utilized for all models to remain consistent in analysis. In addition to the identified models, the previously established ATLAS score for predicting severe CDI was included as a control for models specific to recurrence vs general disease severity (Table 1).^20^

Clinical data were gathered using the University of Virginia Clinical Data Warehouse, a database containing billing/coding, clinical, pharmacy, and laboratory data. Baseline features were defined as the closest available measurement within ±48 hours of *C. difficile* infection diagnosis (defined by the date/time of index positive *C. difficile* PCR specimen collection). For cases with multiple available laboratory measurements, maximum white blood cell count (WBC), creatinine, and minimum albumin measurements were used. For the Eyre model,^18^ all patients received 1 point for ≥3 unformed stools/day as this was a criterion for testing based on UVA diagnostic stewardship practices.^24^ Past gastroenterology admission and C-reactive protein measurements (Eyre score) and presence/absence ≥10 unformed bowel movements/day (D’Agostino) could not be reliably collected. These were imputed as 0 added points when missing. For the IDSA/SHEA Recurrence risk stratification, one point was empirically assigned for each risk factor for recurrent CDI from the Updated 2017 IDSA/SHEA Guidelines. IDSA/SHEA Guidelines include age ≥65 years, a recurrent CDI episode within the last 6 months, immunocompromised host, and severe CDI on presentation which included the measures of white blood cell count ≥15,000 cells/ml, serum creatinine level >1.5mg/dl, or signs of fulminancy (hypotension, shock, ileus, or megacolon).^8,25^

An immunocompromised host was defined as active (administered during hospitalization) receipt of immunosuppressant medications (≥60 mg oral daily prednisone or equivalent systemic corticosteroid, azathioprine, rapamycin derivatives, cyclosporine, tacrolimus, or mycophenolate) and/or chemotherapy (Supplementary Table 1).^19^ Antimotility medications were defined as loperamide, diphenoxylate, oral opium, or bismuth subsalicylate (receipt within 7 days preceding CDI diagnosis).

### Data analysis

The area under the receiver operating characteristic curve (AUROC) for each model were calculated from these score-specific diagnostic test summary indices. The Youden Index (sensitivity + specificity -1) was calculated as an overall measure of diagnostic effectiveness and as one method to identify optimal cutoffs that balance sensitivity and specificity.

DeLong’s test of variance was used to calculate two-sided statistical comparisons of the highest performing model AUROC against each of the others. This was done using correlated AUROC curves. The significance of the AUROC was calculated using only using the patients with complete data for both models being compared. Analyses were performed using statistical software R, version 4.2.3 (R Core Team, Vienna, Austria) and R packages: dpylr,^26^ comorbidity,^27^ ROCit,^28^ pROC,^29^ and PRROC.^30^

## Results

### Study population

1,519 hospitalized cases of *C. difficile* infection among 1,302 individuals were identified between January 2014 and April 2021 with available PCR cycle threshold, white blood cell count, and creatinine measurements to calculate risk scores. Baseline characteristics are shown in Table 2.

**Table 2.** Study population baseline characteristics

Feature	No recurrence (N = 1174)	Recurrence (N = 345)	Overall (N = 1519)
Age			
Mean (SD)	60.4 (16.7)	60.6 (15.9)	60.4 (16.5)
Median [Min, Max]	63.0 [18.0, 91.0]	63.0 [18.0, 91.0]	63.0 [18.0, 91.0]
Sex			
Female	590 (50.1%)	161 (46.7%)	751 (49.4%)
Male	584 (49.7%)	184 (53.3%)	768 (50.6%)
Patient race			
African American	234(19.9%)	48 (13.9%)	282 (18.6%)
Asian	8 (0.5%)	2 (0.6%)	10 (0.7%)
Other	13 (1.3%)	5 (1.4%)	20 (1.3%)
White or Caucasian	917 (78.1%)	290 (84.1%)	1207 (79.5%)
Ethnic group			
Hispanic	15 (1.3%)	1 (0.2%)	36 (1.1%)
Non-Hispanic	1154 (93.5%)	476 (96.0%)	3123 (93.9%)
Patient Refused	4 (0.3%)	1 (0.3%)	5 (0.3%)
Patient Unavailable	1 (0.1%)	0 (0%)	1 (0.1%)
Duration (days) of hospitalization prior to CDI episode			
Mean (SD)	7.67 (10.5)	8.95 (13.1)	7.96 (11.1)
Median [Min, Max]	5.00 [0, 79.0]	5.00 [0, 134]	5.00 [0, 134]
Healthcare facility-onset CDI			
Community-Onset CDI	336 (28.6%)	111 (32.2%)	447 (29.4%)
Community-Onset Healthcare Facility Associated CDI	173 (14.7%)	68 (19.7%)	241 (15.9%)
Healthcare Facility-Onset CDI	665 (56.6%)	166 (48.1%)	831 (54.7%)
Inpatient mortality			
No	1070 (91.1%)	336 (97.4%)	1406 (92.6%)
Yes	104 (8.9%)	9 (2.6%)	113 (7.4%)
In hospital mortality attributable to CDI			
No	1091 (92.9%)	343 (99.4%)	1434 (94.4%)
Yes	83 (7.1%)	2 (0.6%)	85 (5.6%)
Ninety day mortality			
No	962 (81.9%)	307 (89.0%)	1269 (83.5%)
Yes	212 (18.1%)	38 (11.0%)	250 (16.5%)
Colectomy or diverting ileostomy after CDI diagnosis			
No	1049 (89.4%)	325 (94.2%)	1374 (90.5%)
Yes	125 (10.6%)	20 (5.8%)	145 (9.5%)

### Recurrent infection

157 recurrent CDI (rCDI) events were identified based on a repeat positive *C. difficile* test alone, using the UVA Health laboratory records. Clinical chart review performed for the remaining 1,362 cases found an additional 50 rCDI events with repeat positive *C. difficile* tests (either from the Care Everywhere^TM^ electronic medical record-sharing feature in Epic^TM^ or textually reported from an outside laboratory within clinician notes), and 138 rCDI events identified based on symptom relapse following anti-CDI treatment requiring retreatment without retesting. No rCDI cases were identified with negative PCR testing. In total, 345/1,519 (22.7%) patients were found to have recurrent CDI within 60 days.

1,211/1,519 (79.7%) of patients had at least one IDSA-defined risk factor for recurrent infection, all of which (recurrent CDI episode within the last 6 months, immunocompromised host, severe CDI on presentation) except one (age ≥65) was more likely among cases with subsequent recurrence. PCR Cycle threshold (Ct) among patients who went on to develop recurrent infection was lower (ie, higher stool organism burden) compared to patients without recurrence (mean PCR Ct 25.7 recurrence vs 26.8 no recurrence; *t*-test *P* = .0003). A frequency table of score distributions is shown in Table 3 and Supplementary Table 2.

**Table 3.** Frequency table for recurrent CDI score distributions

Score (numerical)	Cobo	Eyre	Larrainzar-Coghen	Reveles	ATLAS	IDSA	D’Agostino Probability	D’Agostino	Cycle Threshold	Cycle Threshold
0	837	9	413	51	30	106	**0.18**	459	<22.1	423
1	286	131	686	65	118	568	**0.24**	81	<25.3	836
2	325	274	358	324	157	668	**0.25**	106	<30.8	1248
3	51	318	58	527	235	159	**0.29**	525		
4	20	337	4	172	274	19	**0.32**	11		
5		212		340	272		**0.35**	165		
6		131		125	215		**0.39**	139		
7		70		15	122		**0.** **45**	33		
8		25			63					
9		9			31					
10		3			2					
No Recurrence (N = 1174): Mean (SD)	0.756 (0.988)	3.69 (1.79)	1.05 (0.820)	3.29 (1.40)	4.32 (2.05)	1.61 (0.814)		0.271 (0.0733)		26.8 (5.21)
Recurrence (N = 345): Mean (SD)	0.817 (0.964)	3.75 (1.66)	1.04 (0.847)	3.40 (1.49)	4.26 (2.02)	1.65 (0.801)		0.269 (0.0741)		25.7 (5.01)
Overall (N = 1519): Mean (SD)	0.770 (0.983)	3.71 (1.76)	1.05 (0.826)	3.32 (1.49)	4.31 (2.04)	1.61 (0.811)		0.271 (0.0735)		26.5 (5.18)

Top: The number of patients were stratified by either numerical, integer-based scores, probability of recurrence according to the D’Agostino Model (in bold), or cycles for each of the recurrence models. Bottom: The mean of value for each model in the populations with no recurrence, recurrence, or overall population. Supplementary Table 2 includes the median.

### Clinical prediction tools

**Figure 1.**
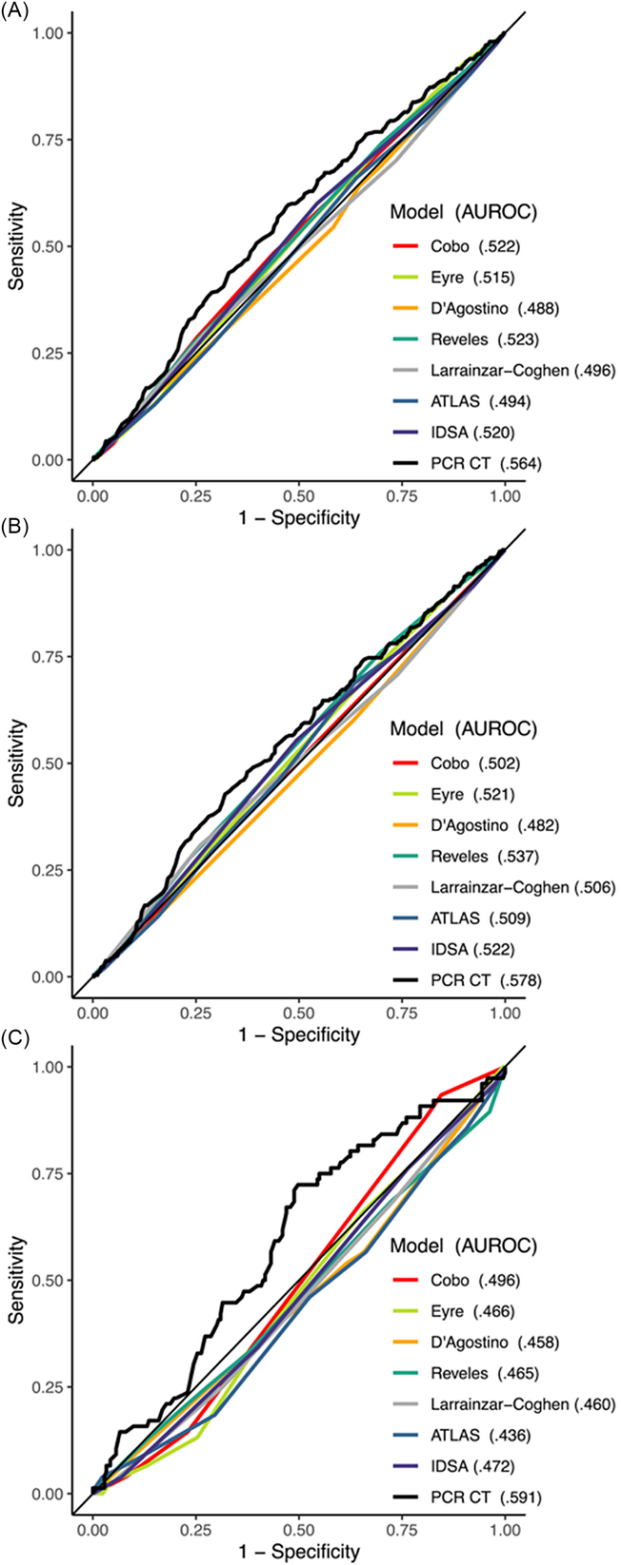
Receiver operator curves with area under the receiver operating characteristic curve (AUROC) for *C. difficile* risk models with the full cohort (1,519 cases) (A), patients with no previous recurrence (1,230 cases) (B), and patients with 1 or more previous recurrent episodes (289 cases) (C). Note: Data was unavailable for some patients. See Table 3 and Supplementary Table 2 for the stratification of the data including the number of missing data points for each model.

AUROCs predicting recurrence of all tools are shown in Figure 1 for all patients (1A), patients with no previous recurrence (1B), and patients with 1 or more previous recurrent episodes (1C). An AUROC of 0.500 represents the prediction ability of random guessing. Each model performed poorly in this validation with the best performing model, Reveles *et al*, having an AUROC of 0.523, 95% CI [0.490, 0.555] when all occurrences were considered. The IDSA guideline recurrence risk criteria (AUROC of 0.520) performed as well as the trained models and 75/345 (21.7%) of patients who developed recurrent CDI had no IDSA-defined recurrence risk factors. The ATLAS score, designed to predict severe outcomes and not recurrence, had a low AUROC of 0.494. The two models which had been previously validated, Cobo *et al* and Larrainzar-Coghen *et al*, performed worse in our validation than in the derivation cohort but similar to previous validation study (Table 1).

**Figure 2.**
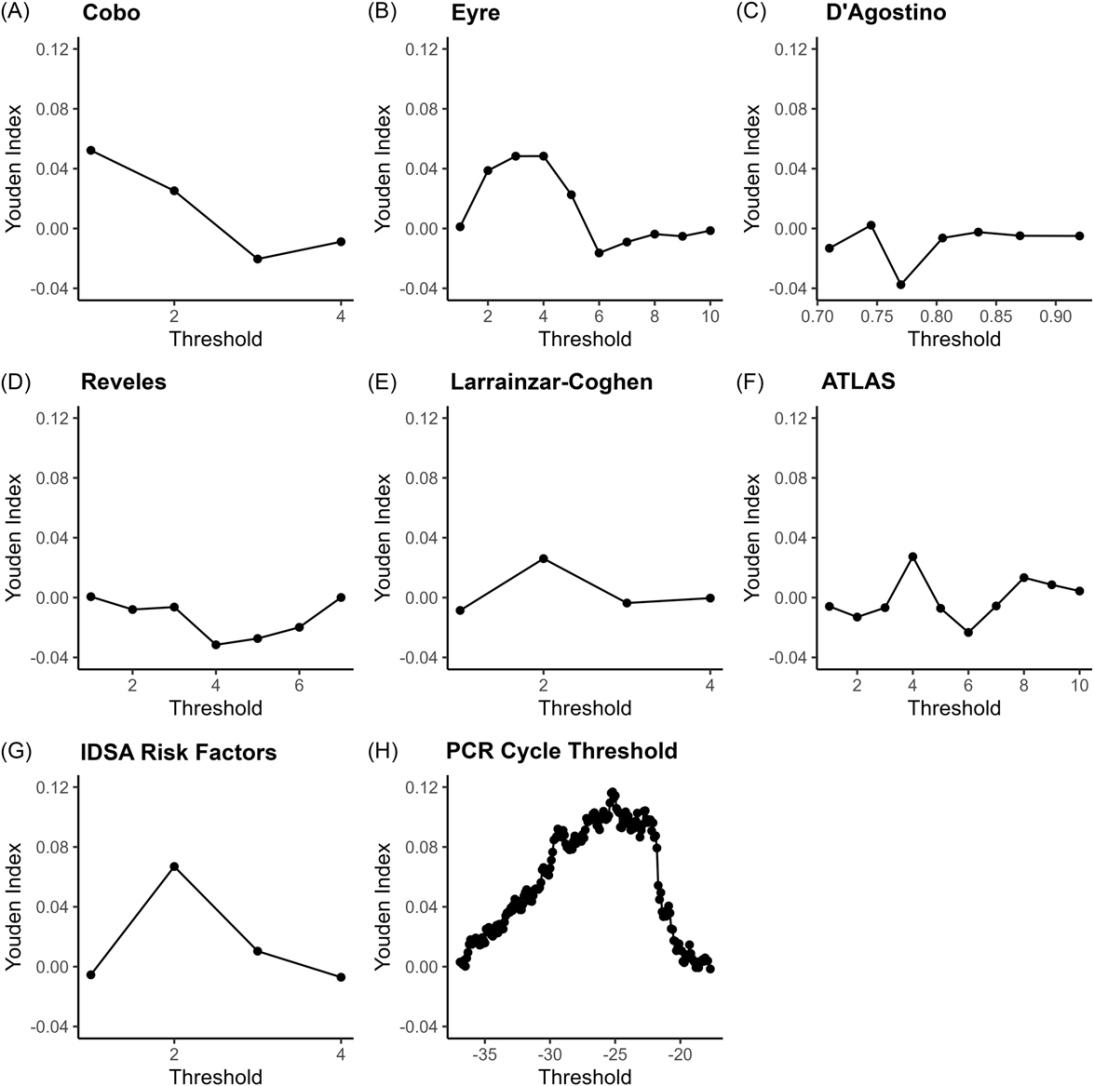
Youden Indices for *C. difficile* Severity Score Cut-offs. Youden Index is equal to 0 for tests with poor diagnostic accuracy, equal to 1 for a perfect test, and assigns equal weight to sensitivity and specificity. The Youden Index and the ideal cutoff may not apply to other patient populations.^31^

PCR Ct had a higher AUROC (0.564) compared to any of the clinical tools, including Reveles *et al* (DeLong’s test of AUROC difference *P* = .082), and IDSA (DeLong’s test of AUROC difference *P* = .052), but AUROC differences were not statistically significant. Median PCR Ct was 25.3 and the positive/negative predictive values at the 1st, 2nd, and 3rd quartile cutoffs (<22.1, <25.3, <30.8) were 0.29/0.80, 0.26/0.82 and 0.23/0.82, respectively. Most tools performed better when only the likelihood of the first recurrence was evaluated (Figure 1B). However, all tools except PCR Ct (AUROC 0.591) preformed worse when only patients with at least one previous recurrence were considered (Figure 1C). Youden index (which maximizes specificity plus sensitivity) curves generated for all cases for each score are shown in Figure 2.

## Discussion

Accurately anticipating risk is a crucial step towards improving access and stewardship of effective new therapies to prevent recurrent CDI, however, predicting future recurrence is a difficult problem. We demonstrate that existing clinical risk scores generalize poorly among a large cohort of hospitalized patients with *C. difficile* infection, especially early in the disease course. The rudimentary methods of generating a scoring systems used by these tools (ie, rounding logistic regression coefficients to integers) may have been inaccurate due to model bias, however, Escobar *et al* strove to generate more robust models using machine learning techniques and concluded that neither existing models nor their own model could be trained to accurately predict recurrent *C. difficile* infection in test patients (maximum achievable out-of-sample validation AUC 0.605).^19^ Other factors to explain poor generalizability include significant *C. difficile* strain variation^32^ or population-level differences (ie, location, socio-economic status, cohort recruitment, etc) between institutions that may influence recurrence risk or unmeasured putative features such as the delayed anti-toxin humoral immune response.^33^

Established risk factors for recurrent infection per recent consensus management guidelines are of marginal value for predicting recurrent *C. difficile* infection, with similar performance to other curated models. Discrepancy between reports of advanced age ≥65 as a positive risk factor for recurrent infection may be due to the competing risk of death within 60 days, which was two times higher among older patients ≥65 years in our cohort (139/685 (20%) patients ≥65 years vs 81/834 (9.7%) <65 years).

A significant hurdle in developing a generalizable and robust prediction tool for clinicians is the inability for the generated models to accurately predict outside of the data on which they were trained. All tools evaluated in this study performed more poorly than in the study in which they were derived. Similarly, when Rossen *et al* ^12^ validated the Cobo and Larrainzar-Coghen models, they observed lower AUROCs, with each model performing worse than random chance. (Table 1) Escobar *et al* ^19^ observed a similar phenomenon in the models which they generated and in a previously generated model. This suggests that prediction tools have been overfitted to the data on which they were trained. A deep neural network with regularization may help to optimize the bias-variance tradeoff while incorporating a higher dimension of inputs to improve prediction. However, it is also possible that the task of accurately predicting future recurrence using clinical observations at the time of index CDI diagnosis is too complex or simply not feasible. Simple measures, such PCR Ct, may be more universally predictive throughout patient populations.

This analysis was intended as a practical assessment of existing tools implemented electronically to predict rCDI and there are important limitations to this analysis. rCDI tools were not validated using the exact same rCDI time frame definitions to which they were trained but, for feasibility reasons, a single rCDI definition was used. For example, the Eyre tool (trained to predict rCDI by 4 months) may perform better predicting late rCDI or relapse. In addition, several potentially important clinical features were omitted from some models because they could not be collected electronically (eg, stool frequency). *C. difficile* misdiagnosis (ie, colonization versus true infection) is a substantial issue in CDI, especially in the context of PCR-based testing and may have confounded both existing tools and our validations.^34^ Recurrent *C. difficile* infection is typically confirmed by repeat testing, which may not be reported in the same electronic medical record or may not be feasible in favor of expediting re-initiation of anti-*C. difficile* treatment (eg, in outpatient settings). To avoid undercapturing outcome events, we defined recurrent CDI broadly to include cases without retesting if symptoms relapsed requiring a new round of anti-*C. difficile* therapy. While our rCDI may have over captured some cases, we believe a symptom-based definition is a pragmatic approach to identify clinically relevant rCDI retrospectively, especially considering that PCR testing often remains positive regardless of rCDI.^35^ Our resulting rate of recurrence (22.7%) approximates rates from prospective trials (∼25%).^25^ 40% of retrospective rCDI events occurred without retesting at the same health system laboratory, underscoring potential flaws in retrospective studies that use test-based rCDI definitions (and thus likely artifactually low rCDI rates) and which could help explain the poor generalizability and performance of existing rCDI tools.

A better, validated prediction tool for recurrence would be helpful to individualize therapeutic approaches to prevent recurrent infection at the time of diagnosis (eg, prioritize fidaxomicin treatment and referral for bezlotoxumab in patients with highest risk for recurrence). Interestingly, of all tools evaluated in this study, PCR Ct had the highest AUROC, similar to at least 4 other studies demonstrating that low CT and/or stool toxin concentration predict rCDI.^36–39^ However, it should be noted that AUROC comparisons between categorical and quasi-continuous measures may not be valid given that scale discretization (of categorical risk scores) may reduce the precision of ROC measurements.^40^ While not a very useful standalone predictor, PCR Ct in a more complex model could increase the reliability of predicting recurrent CDI. Unlike the other biomarkers identified, PCR Ct is readily accessible (PCR is now used by >70% of US hospitals to diagnose CDI),^41^ for any positive *C. difficile* PCR^39^ but Ct data are not traditionally reported to clinicians.

Future studies should carefully plan analysis to avoid common issues (eg, overfitting) and may benefit from considering novel biomarkers and/or higher-dimensional models that could augment or replace existing tools that underperform.

## Supporting information

Boone et al. supplementary material Boone et al. supplementary material

## Supplementary material

The supplementary material for this article can be found at https://doi.org/10.1017/ice.2024.75.

## References

[1] Knight DR , Imwattana K , Collins DA , et al. Genomic epidemiology and transmission dynamics of recurrent Clostridioides difficile infection in Western Australia. Eur J Clin Microbiol Infect Dis 2023;42:607–619. 36940050 10.1007/s10096-023-04569-x PMC10105659

[2] Naz F , Petri WA. Host immunity and immunization strategies for Clostridioides difficile infection. Clin Microbiol Rev 2023;36:e0015722. 37162338 10.1128/cmr.00157-22 PMC10283484

[3] Cornely OA , Crook DW , Esposito R , et al. Fidaxomicin versus vancomycin for infection with Clostridium difficile in Europe, Canada, and the USA: a double-blind, non-inferiority, randomised controlled trial. Lancet Infect Dis 2012;12:281–289. 22321770 10.1016/S1473-3099(11)70374-7

[4] Wilcox MH , Gerding DN , Poxton IR , et al. Bezlotoxumab for prevention of recurrent Clostridium difficile infection. New Engl J Med 2017;376:305–317. 28121498 10.1056/NEJMoa1602615

[5] Kelly CR , Khoruts A , Staley C , et al. Effect of fecal microbiota transplantation on recurrence in multiply recurrent Clostridium difficile infection: a randomized trial. Ann Intern Med 2016;165:609–616. 27547925 10.7326/M16-0271 PMC5909820

[6] FDA News. FDA Approves First Orally Administered Fecal Microbiota Product for the Prevention of Recurrence of Clostridioides difficile Infection. U.S. Food & Drug Administration. https://www.fda.gov/news-events/press-announcements/fda-approves-first-orally-administered-fecal-microbiota-product-prevention-recurrence-clostridioides. Published 2023. Accessed 2024

[7] Jiang Y , Sarpong EM , Sears P , Obi EN. Budget impact analysis of fidaxomicin versus vancomycin for the treatment of Clostridioides difficile infection in the United States. Infect Dis Ther 2022;11:111–126. 34292496 10.1007/s40121-021-00480-0 PMC8847493

[8] Johnson S , Lavergne V , Skinner AM , et al. Clinical practice guideline by the infectious diseases society of America (IDSA) and society for healthcare epidemiology of America (SHEA): 2021 focused update guidelines on management of Clostridioides difficile infection in adults. Clin Infect Dis 2021;73:e1029–44. 34164674 10.1093/cid/ciab549

[9] Lee Y , Lim WI , Bloom CI , Moore S , Chung E , Marzella N. Bezlotoxumab (Zinplava) for Clostridium difficile infection: the first monoclonal antibody approved to prevent the recurrence of a bacterial infection. P T Peer-reviewed J Formulary Manage 2017;42:735–738. PMC5720485 29234211

[10] Cruz MP. Fidaxomicin (dificid), a novel oral macrocyclic antibacterial agent for the treatment of Clostridium difficile-associated diarrhea in adults. P T Peer-reviewed J Formulary Manage 2012;37:278–281. PMC3411227 22876085

[11] Cobo J , Merino E , Martínez C , et al. Prediction of recurrent clostridium difficile infection at the bedside: the GEIH-CDI score. Int J Antimicrob Ag 2018;51:393–398. 10.1016/j.ijantimicag.2017.09.010 28939450

[12] van Rossen TM , van Dijk LJ , Heymans MW , Dekkers OM , Vandenbroucke-Grauls CMJE , van Beurden YH. External validation of two prediction tools for patients at risk for recurrent Clostridioides difficile infection. Ther Adv Gastroenter 2021;14:1756284820977385. 10.1177/1756284820977385 PMC7797589 33456500

[13] Eyre DW , Walker AS , Wyllie D , et al. Predictors of first recurrence of Clostridium difficile infection: implications for initial management. Clin Infect Dis 2012;55:S77–S87. 22752869 10.1093/cid/cis356 PMC3388024

[14] D’Agostino RB , Collins SH , Pencina KM , Kean Y , Gorbach S. Risk estimation for recurrent Clostridium difficile infection based on clinical factors. Clin Infect Dis 2014;58:1386–1393. 24599770 10.1093/cid/ciu107

[15] Reveles KR , Mortensen EM , Koeller JM , et al. Derivation and validation of a Clostridium difficile infection recurrence prediction rule in a national cohort of Veterans. Pharmacother J Hum Pharmacol Drug Ther 2018;38:349–356. 10.1002/phar.2088 PMC5867250 29393522

[16] Larrainzar-Coghen T , Rodriguez-Pardo D , Puig-Asensio M , et al. First recurrence of Clostridium difficile infection: clinical relevance, risk factors, and prognosis. Eur J Clin Microbiol Infect Dis 2016;35:371–378. 26753991 10.1007/s10096-015-2549-9

[17] Hu MY , Katchar K , Kyne L , et al. Prospective derivation and validation of a clinical prediction rule for recurrent Clostridium difficile infection. Gastroenterology 2009;136:1206–1214. 19162027 10.1053/j.gastro.2008.12.038

[18] Zilberberg MD , Reske K , Olsen M , Yan Y , Dubberke ER. Development and validation of a recurrent Clostridium difficile risk-prediction model. J Hosp Med 2014;9:418–423. 24700708 10.1002/jhm.2189

[19] Escobar GJ , Baker JM , Kipnis P , et al. Prediction of recurrent Clostridium difficile infection using comprehensive electronic medical records in an integrated healthcare delivery system. Infect Control Hosp Epidemiol 2017;38:1196–1203. 28835289 10.1017/ice.2017.176 PMC6008100

[20] Miller MA , Louie T , Mullane K , et al. Derivation and validation of a simple clinical bedside score (ATLAS) for Clostridium difficile infection which predicts response to therapy. Bmc Infect Dis 2013;13:148. 23530807 10.1186/1471-2334-13-148 PMC3618004

[21] Madden GR , Rigo I , Boone R , et al. Novel biomarkers, including tcdB PCR cycle threshold, for predicting recurrent Clostridioides difficile infection. Infect Immun 2023;91:e00092–23. 36975808 10.1128/iai.00092-23 PMC10112139

[22] Madden GR , Enfield KB , Sifri CD. Patient outcomes with prevented versus negative Clostridioides difficile tests using a computerized clinical decision support (CCDS) tool. Open Forum Infect Dis 2020;7:ofaa094. 32328506 10.1093/ofid/ofaa094 PMC7166115

[23] Madden GR , Petri WA , Costa DVS , Warren CA , Ma JZ , Sifri CD. Validation of clinical risk models for Clostridioides difficile-attributable outcomes. Antimicrob Agents Ch 2022;66:e00676–22. 10.1128/aac.00676-22 PMC9295569 35727061

[24] Madden GR , Mesner IG , Cox HL , et al. Reduced Clostridium difficile tests and laboratory-identified events with a computerized clinical decision support tool and financial incentive. Infect Control Hosp Epidemiol 2018;39:737–740. 29644943 10.1017/ice.2018.53 PMC6088779

[25] McDonald LC , Gerding DN , Johnson S , et al. Clinical practice guidelines for Clostridium difficile infection in adults and children: 2017 update by the infectious diseases society of America (IDSA) and society for healthcare epidemiology of America (SHEA). Clin Infect Dis 2018;66:e1–e48. 29462280 10.1093/cid/cix1085 PMC6018983

[26] Wickham H , Francois R , Henry L , Muller K. dplyr: A Grammar of Data Manipulation. R package version 1.0.7. https://CRAN.R-project.org/package=dplyr. Published 2022. Accessed 2022

[27] Gutiérrez-Sacristán A , Bravo À , Giannoula A , Mayer MA , Sanz F , Furlong LI. comoRbidity: an R package for the systematic analysis of disease comorbidities. Bioinf 2018;34:3228–3230. 10.1093/bioinformatics/bty315 PMC6137966 29897411

[28] Khan RA. ROCit: An R Package for Performance Assessment of Binary Classifier with Visualization. https://CRAN.R-project.org/package=ROCit. Published 2020. Accessed 2024

[29] Robin X , Turck N , Hainard A , et al. pROC: an open-source package for R and S+ to analyze and compare ROC curves. BMC Bioinf 2011;12:77. 10.1186/1471-2105-12-77 PMC3068975 21414208

[30] Keilwagen J , Grosse I , Grau J. Area under precision-recall curves for weighted and unweighted data. Plos One 2014;9:e92209. 24651729 10.1371/journal.pone.0092209 PMC3961324

[31] Schisterman EF , Perkins NJ , Liu A , Bondell H. Optimal cut-point and its corresponding youden index to discriminate individuals using pooled blood samples. Epidemiol 2005;16:73–81. 10.1097/01.ede.0000147512.81966.ba 15613948

[32] Walker AS , Eyre DW , Wyllie DH , et al. Relationship between bacterial strain type, host biomarkers, and mortality in Clostridium difficile infection. Clin Infect Dis Off Publ Infect Dis Soc Am 2013;56:1589–1600. 10.1093/cid/cit127 PMC3641870 23463640

[33] Gupta SB , Mehta V , Dubberke ER , et al. Antibodies to Toxin B are protective against Clostridium difficile infection recurrence. Clin Infect Dis 2016;63:730–734. 27365387 10.1093/cid/ciw364

[34] Polage CR , Gyorke CE , Kennedy MA , et al. Overdiagnosis of Clostridium difficile infection in the molecular test era. Jama Intern Med 2015;175:1792–1801. 26348734 10.1001/jamainternmed.2015.4114 PMC4948649

[35] Saha S , Yadav D , Pardi R , Patel R , Khanna S , Pardi D. Kinetics of polymerase chain reaction positivity in patients with Clostridioides difficile infection. Ther Adv Gastroenterol 2021;14:17562848211050444. 10.1177/17562848211050443 PMC8504224 34646361

[36] Alonso CD , Kelly CP , Garey KW , et al. Ultrasensitive and quantitative toxin measurement correlates with baseline severity, severe outcomes, and recurrence among hospitalized patients with Clostridioides difficile infection. Clin Infect Dis 2021;74:ciab826. 10.1093/cid/ciab826 PMC9258941 34537841

[37] Origüen J , Orellana MÁ , Fernández-Ruiz M , et al. Toxin B PCR amplification cycle threshold adds little to clinical variables for predicting outcomes in Clostridium difficile infection: a retrospective cohort study. J Clin Microbiol 2019;57:e01125–18. 10.1128/JCM.01125-18 PMC6355545 30463889

[38] Kociolek LK , Palac HL , Patel SJ , Shulman ST , Gerding DN. Risk factors for recurrent Clostridium difficile infection in children: a nested case-control study. J Pediatr 2015;167:384–389. 26001313 10.1016/j.jpeds.2015.04.052

[39] Sandora TJ , Kociolek LK , Williams DN , et al. Baseline stool toxin concentration is associated with risk of recurrence in children with Clostridioides difficile infection. Infect Control Hosp Epidemiol 2023;44:1403–1409. 36624698 10.1017/ice.2022.310 PMC10330943

[40] Wagner RF , Beiden SV , Metz CE. Continuous versus categorical data for ROC analysis some quantitative considerations. Acad Radiol 2001;8:328–334. 11293781 10.1016/S1076-6332(03)80502-0

[41] Guh AY , Mu Y , Winston LG , et al. Trends in U.S. burden of Clostridioides difficile infection and outcomes. N Engl J Med 2020;382:1320–1330. 32242357 10.1056/NEJMoa1910215 PMC7861882

