# Supplementary material for: Validation of clinical risk tools for recurrent Clostridioides difficile Infection
Source: Infect Control Hosp Epidemiol. Author manuscript; Available in PMC 2025 Nov 9. (PMC11518676; doi:10.1017/ice.2024.75)
Supplement: Supplementary Material [file NIHMS2005366-supplement-Supplementary_Material.pdf]

Supplementary Information

Predicting *Clostridioides difficile* Infection Outcomes with Explainable Machine Learning

Gregory R. Madden MD MSDS, Rachel H. Boone, Emmanuel Lee BSN RN, Costi D. Sifri MD, William A. Petri Jr MD PhD

Table of Contents:

Supplementary Table 1: List of ICD-10 Codes for Ileus 2
Supplementary Table 2: List of Chemotherapies 3
Supplementary Figure 1: Deep Neural Network Model Connectivity Graph 4
Supplementary Figure 2: Deep Neural Network Model Connectivity Graph 5

Supplementary Table 1: List of ICD-10 Codes for Ileus	
**1**	K56.609 - Unspecified intestinal obstruction, unspecified as to partial versus complete obstruction
**2**	K56.0 - Paralytic ileus
**3**	K56.7 - Ileus, unspecified
**4**	K56.600 - Partial intestinal obstruction, unspecified as to cause
**5**	K56.2 - Volvulus
**6**	K56.699 - Other intestinal obstruction unspecified as to partial versus complete obstruction
**7**	K56.41 - Fecal impaction
**8**	K56.69 - Other intestinal obstruction
**9**	K56.1 - Intussusception
**10**	K56.690 - Other partial intestinal obstruction

International Classification of Diseases 10 (ICD-10) codes used to define the presence of early ileus (at least 1 billing code documented within 48 hours of diagnosis) among patients with *C. difficile* infection. Use of ICD codes allowed the presence of ileus (used in the severity model by Belmares et al. and recommended by the IDSA/SHEA clinical management guidelines for risk stratifying *C. difficile* infection) to be gathered electronically from the electronic medical record.

Supplementary Table 2: List of Chemotherapies					
**1**	Abraxane	**29**	Doxifluridine	**57**	Oxaliplatin
**2**	Actinomycin	**30**	Doxorubicin	**58**	Paclitaxel
**3**	Alitretinoin	**31**	Epirubicin	**59**	Pemetrexed
**4**	All-trans retinoic acid	**32**	Epothilone	**60**	Pipobroman
**5**	Altretamine	**33**	Erlotinib	**61**	Ranimustine
**6**	Azacitidine	**34**	Etoposide	**62**	Romidepsin
**7**	Azathioprine	**35**	Exatecan	**63**	Semustine
**8**	Belotecan	**36**	Fluorouracil	**64**	Streptozotocin
**9**	Bendamustine	**37**	Fotemustine	**65**	Tafluposide
**10**	Bexarotene	**38**	Gefitinib	**66**	Taxotere
**11**	Bleomycin	**39**	Gemcitabine	**67**	Temozolomide
**12**	Bortezomib	**40**	Gimatecan	**68**	Tesetaxel
**13**	Busulfan	**41**	Hydroxyurea	**69**	Teniposide
**14**	Cabazitaxel	**42**	Idarubicin	**70**	Thiotepa
**15**	Camptothecin	**43**	Ifosfamide	**71**	Tioguanine
**16**	Carboplatin	**44**	Imatinib	**72**	Topotecan
**17**	Carboquone	**45**	Irinotecan	**73**	Treosulfan
**18**	Carmustine	**46**	Ixabepilone	**74**	Tretinoin
**19**	Capecitabine	**47**	Larotaxel	**75**	Triaziquone
**20**	Cisplatin	**48**	Lomustine	**76**	Triethylenemelamine
**21**	Chlorambucil	**49**	Melphalan	**77**	Valrubicin
**22**	Chlormethine	**50**	Mercaptopurine	**78**	Vemurafenib
**23**	Chlorozotocin	**51**	Methotrexate	**79**	Vinblastine
**24**	Cyclophosphamide	**52**	Mitobronitol	**80**	Vincristine
**25**	Cytarabine	**53**	Mitomycin	**81**	Vindesine
**26**	Dacarbazine	**54**	Mitoxantrone	**82**	Vinorelbine
**27**	Daunorubicin	**55**	Nimustine	**83**	Vismodegib
**28**	Docetaxel	**56**	Nitrosoureas	**84**	Vorinostat

Chemotherapeutic drug names used to define the “Active Chemotherapy” feature (administered during same hospitalization), based on the *C. difficile* severity scoring tool developed by Eyre et al.

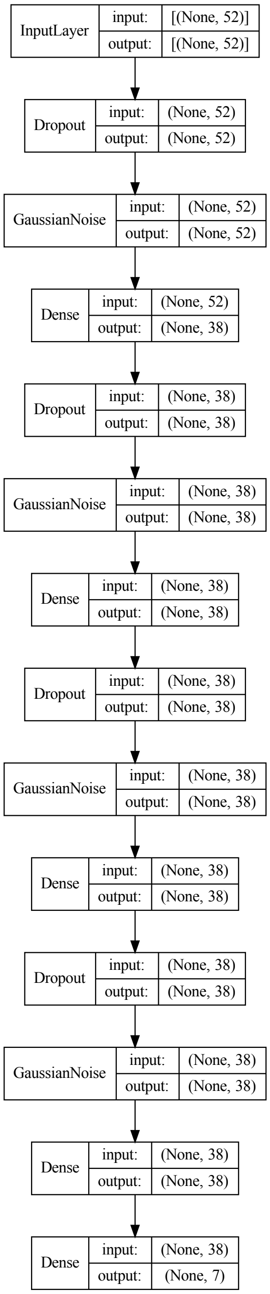

Supplementary Figure 1: Deep Neural Network Model Connectivity Graph
A network connectivity graph demonstrating the deep neural network structure, including 52 total feature inputs, 4 hidden layers (each with 38 neurons, determined using Bayesian inference), and a 7 desirability of outcome ranking output layer (with softmax function). Dropout (10%) and Gaussian noise (0.2 standard deviations) were utilized for regularization.

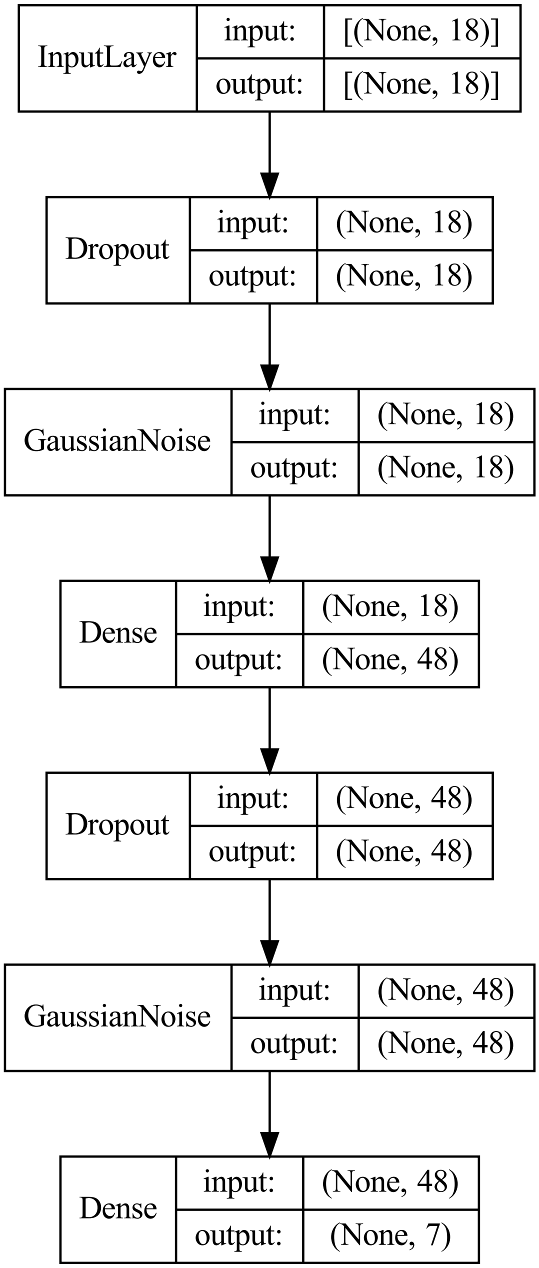

Supplementary Figure 2: Deep Neural Network Model Connectivity Graph
A network connectivity graph demonstrating the reduced, shallow neural network structure with 18 total feature inputs, a single hidden layer (with 48 neurons), and the 7 desirability of outcome ranking output layer (with softmax function). Dropout (10%) and Gaussian noise (0.2 standard deviations) were utilized for regularization.
