# Supplementary material for: Validation of clinical risk tools for recurrent Clostridioides difficile infection
Source: Infect Control Hosp Epidemiol. 2024 May 9;45(9):1064–72. doi: 10.1017/ice.2024.75 (PMC11518676; doi:10.1017/ice.2024.75)
Supplement: Supplementary file 1 [file S0899823X24000758sup001.docx]

**Supplementary Material for:** 

**Validation of Clinical Risk Models for Recurrent** ***Clostridioides difficile*** **Infection** 

*Rachel H. Boone,*^1^ *Emmanuel Lee BSN RN,*^ 2^ *William A. Petri Jr MD, Ph.D.,*^*1,3*^ *Gregory R. Madden MD, MSDS*^*1,3*^***

^1^Department of Microbiology, Immunology, and Cancer Biology, University of Virginia, Charlottesville, VA, USA

^2^University of Virginia School of Medicine, Charlottesville, VA, USA

^3^Division of Infectious Diseases & International Health, Department of Medicine, University of Virginia School of Medicine, Charlottesville, VA, USA

*	Corresponding Author: Gregory R. Madden, Division of Infectious Diseases & International Health, University of Virginia School of Medicine, P.O. Box 800473, Charlottesville, VA  22908-0473 USA, phone: (434) 243-8898, fax: (434) 924-1225 email: [grm7q@virginia.edu](mailto:grm7q@virginia.edu)

| **Supplementary Table 1: List of Chemotherapies** |  |  |  |  |  |
| --- | --- | --- | --- | --- | --- |
| **1** | Abraxane | **29** | Doxifluridine | **57** | Oxaliplatin |
| **2** | Actinomycin | **30** | Doxorubicin | **58** | Paclitaxel |
| **3** | Alitretinoin | **31** | Epirubicin | **59** | Pemetrexed |
| **4** | All-trans retinoic acid | **32** | Epothilone | **60** | Pipobroman |
| **5** | Altretamine | **33** | Erlotinib | **61** | Ranimustine |
| **6** | Azacitidine | **34** | Etoposide | **62** | Romidepsin |
| **7** | Azathioprine | **35** | Exatecan | **63** | Semustine |
| **8** | Belotecan | **36** | Fluorouracil | **64** | Streptozotocin |
| **9** | Bendamustine | **37** | Fotemustine | **65** | Tafluposide |
| **10** | Bexarotene | **38** | Gefitinib | **66** | Taxotere |
| **11** | Bleomycin | **39** | Gemcitabine | **67** | Temozolomide |
| **12** | Bortezomib | **40** | Gimatecan | **68** | Tesetaxel |
| **13** | Busulfan | **41** | Hydroxyurea | **69** | Teniposide |
| **14** | Cabazitaxel | **42** | Idarubicin | **70** | Thiotepa |
| **15** | Camptothecin | **43** | Ifosfamide | **71** | Tioguanine |
| **16** | Carboplatin | **44** | Imatinib | **72** | Topotecan |
| **17** | Carboquone | **45** | Irinotecan | **73** | Treosulfan |
| **18** | Carmustine | **46** | Ixabepilone | **74** | Tretinoin |
| **19** | Capecitabine | **47** | Larotaxel | **75** | Triaziquone |
| **20** | Cisplatin | **48** | Lomustine | **76** | Triethylenemelamine |
| **21** | Chlorambucil | **49** | Melphalan | **77** | Valrubicin |
| **22** | Chlormethine | **50** | Mercaptopurine | **78** | Vemurafenib |
| **23** | Chlorozotocin | **51** | Methotrexate | **79** | Vinblastine |
| **24** | Cyclophosphamide | **52** | Mitobronitol | **80** | Vincristine |
| **25** | Cytarabine | **53** | Mitomycin | **81** | Vindesine |
| **26** | Dacarbazine | **54** | Mitoxantrone | **82** | Vinorelbine |
| **27** | Daunorubicin | **55** | Nimustine | **83** | Vismodegib |
| **28** | Docetaxel | **56** | Nitrosoureas | **84** | Vorinostat |

| Supplementary Table 2: Frequency Table for Recurrent CDI Score Distributions Continued |  |  |  |  |  |  |  |  |
| --- | --- | --- | --- | --- | --- | --- | --- | --- |
|  | **Score (numerical)** |  |  |  |  |  | **Probability** | **Cycles** |
|  | Cobo | Eyre | Larrainzar-Coghen | Reveles | ATLAS | IDSA | DAgostino | CT |
| No Recurrence (N=1174): Mean (SD) | 0.756 (0.988) | 3.69 (1.79) | 1.05 (0.820) | 3.29 (1.40) | 4.32 (2.05) | 1.61 (0.814) | 0.271 (0.0733) | 0.271 (0.0733) |
| Recurrence (N=345): Mean (SD) | 0.817 (0.964) | 3.75 (1.66) | 1.04 (0.847) | 3.40 (1.49) | 4.26 (2.02) | 1.65 (0.801) | 0.269 (0.0741) | 0.269 (0.0741) |
| Overall (N=1519): Mean (SD) | 0.770 (0.983) | 3.71 (1.76) | 1.05 (0.826) | 3.32 (1.49) | 4.31 (2.04) | 1.61 (0.811) | 0.271 (0.0735) | 0.271 (0.0735) |
| No Recurrence (N=1174): Median [Min, Max] | 0 [0, 4.00] | 4.0 [0, 10.0] | 1.00 [0, 4.00] | 3.00 [0, 7.00] | 4.00 [0, 10.0] | 2.00 [0, 4.00] | 0.290 [0.180, 0.450] | 25.7 [17.7, 37.0] |
| Recurrence (N=345): Median [Min, Max] | 0 [0, 4.00] | 4.00 [0, 9.00] | 1.00 [0, 3.00] | 3.00 [0, 7.00] | 4.00 [0, 9.0] | 2.00 [0, 4.00] | 0.290 [0.180, 0.450] | 24.2 [17.8, 36.9] |
| Overall (N=1519): Median [Min, Max] | 0 [0, 4.00] | 4.00 [0, 10.0] | 1.00 [0, 4.00] | 3.00 [0, 7.00] | 4.00 [0, 10.0] | 2.00 [0, 4.00] | 0.290 [0.180, 0.450] | 25.3 [17.7, 37.0] |
| The mean and median for each model in the No Recurrence, Recurrence, and Overall populations. |  |  |  |  |  |  |  |  |
